# Two new gene clusters involved in the degradation of plant cell wall from the fecal microbiota of Tunisian dromedary

**DOI:** 10.1371/journal.pone.0194621

**Published:** 2018-03-30

**Authors:** Rihab Ameri, Elisabeth Laville, Gabrielle Potocki-Véronèse, Sahar Trabelsi, Monia Mezghani, Fatma Elgharbi, Samir Bejar

**Affiliations:** 1 Laboratoire de Biotechnologie Microbienne et d'Ingénierie des Enzymes (LBMIE), Centre of Biotechnology of Sfax (CBS), University of Sfax, Sfax, Tunisia; 2 LISBP, CNRS, INRA, INSAT, Université de Toulouse, Toulouse, France; Chang Gung University, TAIWAN

## Abstract

Dromedaries are capable of digesting plant cell wall with high content of lignocellulose of poor digestibility. Consequently, their intestinal microbiota can be a source of novel carbohydrate-active enzymes (CAZymes). To the best of our knowledge, no data are available describing the biochemical analysis of enzymes in dromedary intestinal microbiota. To investigate new hydrolytic enzymes from the dromedary gut, a fosmid library was constructed using metagenomic DNA from feces of non-domestic adult dromedary camels living in the Tunisian desert. High-throughput functional screening of 13756 clones resulted in 47 hit clones active on a panel of various chromogenic and non-chromogenic glycan substrates. Two of them, harboring multiple activities, were retained for further analysis. Clone 26H3 displayed activity on AZO-CM-cellulose, AZCL Carob galactomannan and Tween 20, while clone 36A23 was active on AZCL carob galactomannan and AZCL barley β-glucan. The functional annotation of their sequences highlighted original metagenomic loci originating from bacteria of the Bacteroidetes/Chlorobi group, involved in the metabolization of mannosides and β-glucans thanks to a complete battery of endo- and exo-acting glycoside hydrolases, esterases, phosphorylases and transporters.

## Introduction

The dromedary camel (*Camelus dromedarius*) is classified as a pseudo-ruminant; its foregut consists of three compartments instead of four in a true ruminant. This animal is known to withstand harsh environmental conditions that are unfavorable to other herbivores. Due to higher cellulolytic activity and longer retention time of solid particles in their digestive tract [1], dromedaries are able to digest plant cell wall carbohydrates with high content of lignocellulose of poor digestibility (spiny brush, halophytes, etc.) much more effectively than other ruminants [2–4]. To perform plant biomass breakdown, they rely on their gut microbial consortium dominated by fiber-degrading bacteria [5]. Functional analysis of the dromedary gut metagenome revealed that carbohydrate metabolism represents the second most abundant subsystem, and that this microbiome contains the highest percentage of glycoside hydrolases compared with other gastrointestinal metagenomes from human, lion, cattle, rabbit, horse, chimpanzee and dog [6]. Consequently, dromedary intestinal microbiota can be a source of novel carbohydrate-active enzymes (CAZymes) [7] responsible for the degradation, modification or synthesis of glycosidic bonds [8]. They have been described to offer attractive opportunities to the animal feed industry including the increase of hemicellulose digestibility in feed stuffs, the improvement of feed conversion efficiency and the mitigation of sanitary problems such as sticky droppings [9, 10].

So far, all the CAZymes used in the feed industry have been discovered by means of classic culture-dependent methods [11]. However, knowledge of gut ecosystems, where most of the microbiota remains unidentified, is largely incomplete [12]. Only less than 1% of CAZyme sequences in databases have indeed been identified from uncultured microorganisms [13], representing between 60% and 80% of bacteria in digestive ecosystems [14]. Accordingly, the main diversity of CAZymes is locked in the uncultured fraction. To access to this fraction, numerous studies have employed metagenomic approaches to discover novel hydrolytic enzymes by the construction of metagenomic libraries from different ecosystems [15, 16]. To our knowledge, our study was the first aimed at identifying functional microbial polysaccharide degrading gene clusters in the dromedary gut microbiome. Indeed, only one study has yet reported functional analysis of the camel rumen maintained under intensive feeding, by determining and analyzing the whole metagenome, without any biochemical investigation [6]. The present study delves into the functional analysis of the fecal metagenome of a non-domestic adult dromedary that had been browsing native plants in the Tunisian desert. We report the construction of a fosmid library from this dromedary fecal sample and its high throughput activity-based screening in order to identify enzymes that are involved in the hydrolysis of cellulosic and hemicellulosic plant biomass. In addition, we scrutinize a pair of new original metagenomic gene clusters whose products could operate synergistically in the degradation of plant cell wall.

## Materials and methods

### Sampling and DNA extraction

A fresh fecal sample from adult wild dromedary of the Tunisian desert was collected in a sterile container and stored at 4°C. Metagenomic DNA was extracted from the sample as previously described [17] with some modifications. Briefly, 0.5 g of fecal sample was mixed with 1.3 mL of lysis buffer supplemented with 5 mg/ml of lysozyme and incubated at 37°C for 10 min. Three cycles of freezing/heating were performed with liquid nitrogen and at 65°C. The mixture was incubated at 37°C with proteinase K and at 65°C with EDTA then centrifuged (3200 g, 10 min). Cell wall debris, denatured proteins, and most polysaccharides were simultaneously removed during DNA extraction with chloroform and precipitation with isopropanol. DNA quantity was measured using a NanoDrop spectrophotometer (Agilent). DNA was stored at -20°C until use.

### Fosmid library construction

The metagenomic library was constructed using the CopyControl™ Fosmid Library Production Kit with a pCC1FOS™ Vector (Epicentre, Madison, Wisconsin, USA). The DNA fragments were separated in 1% low melting point agarose gel in 1×Tris-Borate-EDTA buffer (89 mM Tris, 89 mM Borate, 2 mM EDTA) at 40 V overnight. Fragments between 30 and 40 Kbp were cut out of the gel and purified by using the freezing/thawing method to isolate DNA from agarose gel in addition to the Gelase supplied with the kit. Purified DNA fragments were end repaired to blunt, 5′-phosphorylated ends, cloned into the pCC1FOS™ Vector and packaged according to the manufacturer’s protocol. For titration, a dilution of phage suspension was used to infect an *Escherichia coli* EPI300-T1R culture in exponential phase. Packaged DNA was stored as a primary library at -80°C in glycerol (20% final concentration) until screening. The whole packaged DNA was then used to infect *Escherichia coli* EPI300-T1R, and the infected clones were plated on Luria-Bertani (LB) Agar containing 12.5 μg/mL chloramphenicol for the picking procedure.

### High throughput functional screening and detection of the activities on crude extract

Colony picking of 13756 recombinant clones was performed using an automated colony picker (Qpix 460, Molecular Devices, Sunnyvale, CA, USA). Colonies were transferred to 384-well plates containing liquid medium (Luria-Bertani, 8% glycerol complemented with 12.5 μg/mL chloramphenicol). After 24 h of growth at 37°C without any agitation, the plates were stored at -80°C. The 13756 clones were screened for fiber degradation activities as previously described [18]. Clones were gridded onto 22 cm x 22 cm bioassay trays containing an LB-agar medium supplemented with 12.5 μg/L chloramphenicol and one of the following chromogenic substrates from Megazyme (AZCL-Galactomannan (Carob), AZCL Xylan (Birchwood), AZCL-Barley β-Glucan, Azo-CM-Cellulose, at final concentration of 0.2% (w/v)) or non-chromogenic (Tween 20 (Polyethylene glycol sorbitan mono laurate, an ester with C12 chain length) at final concentration of 1% (w/v)), using a K2 automated plate replication system (K Biosystem, Basildon, UK). The Q trays were incubated at 37°C from 1 to 10 days, depending on the time necessary to visualize the activities of hit clones. It was checked that the EPI300 strain harboring the empty pCC1FOS vector was unable to react on these media. The positive clones were visually detected by the presence of a blue halo resulting from the production of soluble oligosaccharides that diffused around the bacterial colonies for AZCL-substrates, the appearance of a discoloration halo around positive clones on Azo- substrates and the appearance of a powdery halo around positive clones on Tween 20. To validate the fosmid library screening, positive clones were picked from the Q Tray and streaked on Petri dishes containing LB and chloramphenicol. For each clone, three isolated colonies were then gridded on omnitrays containing the same medium used for the primary screening.

In order to quantify the enzymatic activities in the crude extract, clones were grown in LB liquid medium overnight at 37°C with shaking at 200 rpm. The cultures of 24 h were centrifuged and pellets were resuspended in phosphate buffer containing 0.5 g/L of lysozyme to obtain a final OD at 600 nm of 80. After incubation at 37°C for one hour, cell lysis was completed with a freeze (-80°C) and thaw (30°C) cycle. Crude cell lysates were centrifuged to remove cell debris and supernatants were collected as crude protein extracts for enzymatic assays. Glycoside Hydrolases activity assays were performed using different substrates from Sigma Chemical Co Ltd: Barley β-glucan (β-1,4–1,3-glucan), CM-cellulose (β-1,4-glucan) and Locust Bean Gum (β-1,4-manan). Reactions were carried out in a phosphate buffer (50mM) pH = 7 by adding 0.5 ml of a 0.2%, 0.4% and 0.5% (w/v) of respectively Barley β-glucan, CM-cellulose and Locust Bean Gum solutions to 0.5 ml of crude cytoplasmic extract. The reaction mixture was incubated for 30 min at 37°C. The amount of reducing sugars released was determined by the dinitrosalicylic acid (DNS) method [19].

Esterase activity was measured by titration of the free fatty acids with 100 mM NaOH using Tributyrine (TC4) and an emulsion of olive oil as substrates. The reaction was performed at 37°C and the pH was adjusted to 8 using a pH-stat unit (METROHM 718).

### Sequencing and functional annotation

Fosmid DNA of selected clones was extracted using the alkaline lysis as previously described [20]. The nucleotide sequences were determined using the MiSeq technology (Illumina MiSeq (San Diego, CA)) allowing the generation of thousands to millions of short sequencing reads. This raw sequence dataset was assembled using the SPAdes Genome Assembler (http://bioinf.spbau.ru/en/spades). The fosmid pCC1FOS sequences were identified and discarded. The Rapid Annotation Server (RAST, http://www.nmpdr.org/FIG/wiki/view.cgi/FIG/RapidAnnotationServer) was used to predict open reading frames from the resulting sequences and unveil the repertoire of predicted genes and gene functions. The CAZy family annotation was based on ORFs sequence similarities with proteins of sequenced genomes using BLASTP analysis against the non-redundant NCBI and the SWISSPROT databases. The CAZy family of the best blast hit of each predicted CAZyme was retrieved by searching its accession number in the CAZy database [8]. Contig sequences were submitted to the DDBJ/EMBL/GenBank databases under accession numbers LT796702 for the clone 36A23 and LT796703 for the clone 26H3.

### Taxonomic assignment of the metagenomic sequences

Two methods were used to perform taxonomic assignment of protein-coding genes. The first was based on ORFs sequence similarities with sequenced genomes, using BLASTX analysis against the non-redundant NCBI database (*E*-value ≤10^−8^, identity ≥ 90%, and query length coverage ≥ 50%). A class, genus or species was assigned to a contig when protein-coding genes of this contig were derived from the same organism. The most probable common ancestor from which the non-assignable contig has evolved was retrieved using MEGAN v.5.10.6 [21] based on BLASTX analysis against non-redundant NCBI.

## Results

### Construction of the fecal metagenomic library

Metagenomic DNA was extracted from the fecal sample of an adult dromedary freely living in Tunisian desert as described above. DNA fragments with an average size of about 30 to 40 kbp were purified and used for the construction of the fosmid library. After the in-vitro packaging and transfection, the library was titrated yielding about 14000 clones and stored until screening. The fosmid contain analysis of several clones showed that almost all clones contained an insert of about 30 kb. The total size of this dromedary fecal library is estimated to be about 420 Mb of DNA. If the average size of a bacterial gene is assumed to be ~ 924 bp [22], then we have the potential to be screen about 450,000 environmental genes.

### Activity-based screening of cellulolytic and hemicellulolytic enzymes

The fosmid library was screened for clone ability to degrade chromogenic substrates which mimic the chemical bonds between carbohydrate components of the plant cell wall. Endo-acting hemicellulases were screened by means of AZCL-Galactomannan (Carob), AZCL-Xylan (Birchwood) and AZCL-Barley-β-Glucan and the endo-acting cellulases were screened using Azo-Carbo-Methyl-Cellulose (AZO-CM-Cellulose) chromogenic substrates. Tween 20 was used for the assays of esterase including carbohydrate esterase which are key enzymes to breakdown hemicellulosic compounds. In total, 54 activities were validated, corresponding to 47 positive clones, meaning that some clones harbored several of the screened activities (Table 1).

**Table 1:** Positive clones obtained by high-throughput screening.

substrate	AZCL-xylan	AZCL Carob galactomannan	AZO-CM Cellulose	AZCL Barley beta glucan	Tween 20
**Activity**	Endo-1,4-β-D-xylanase Xylosyl chains	Endo-1,4-β-D-mannanase mannosyl chain ramified with galactosyl residues	Endo-1,4-β-D-glucanase (cellulase)	β-glucanase Endo-1,4-β-D-glucanase (cellulase) and Endo-(1,4)-(1,3)- β-D-glucanase (lichenase)	Esterases
**Number of hit clones**	0	20	17	5	12
**Hit rate**	0	0.08%	0.12%	0.036%	0.09%

Respectively, 17, 20 and 5 hits were found on AZO-CM-Cellulose, AZCL-Galactomannan and AZCL-Barley-β-Glucan. Twelve clones were active on Tween 20. No hit was found on AZCL-Xylan. Among positive clones, two (26H3 and 36A23) displaying multiple activities were further analyzed. Indeed, the first clone (26H3) showed activity on AZO-CM cellulose, AZCL Carob galactomannan, and Tween 20 while the second (36A23) was active on AZCL carob galactomannan and AZCL barley β-glucan (Table 2).

**Table 2:** Activities shown by 26H3 and 36A23 clones.

Clones	AZO-CM Cellulose	AZCL Carob galactomannan	AZCL Barley Beta glucan	AZCL Xyloglucan	AZCL-HE Cellulose	Tween 20
Clone 26H3	+	+	-	-	-	+
Clone 36A23	-	+	+	-	-	-

Furthermore, liquid assays of the different activities on the cytoplasmic extracts of these clones (Table 3) confirm the presence of the activities revealed on high throughput screening.

**Table 3:** Activities detected in the cytoplasmic extracts of clones 26H3 and 36A23.

Substrate	Activity (U/ml)	
		26H3	36A23
Locust Bean Gum (β-1,4-manan)	0.628	0.271
CM-cellulose (β-1,4-glucan)	0.159	ND
Barleyβ-glucan (β-1,4–1,3-glucan)	ND	0.246
Tributyrine (TC4)	70	60.4
Olive oil emulsion	ND	ND

ND: non detected

### Sequencing and annotation

Sequencing of fosmid DNA from the selected clones, 26H3 and 36A23, was performed with the MiSeq (Illumina MiSeq (San Diego, CA)). Read assembly resulted in one large contig of 24131 bp for 26H3 (Accession number: LT796703) and 31146 bp for 36A23 (Accession number: LT796702). Gene prediction of resulting contigs showed that the metagenomic inserts contained 20 ORFs and 29 ORFs respectively for 26H3 and 36A23.

The taxonomic assignment of the predicted genes based on sequence similarities with the non-redundant (NR) protein sequences database of the NCBI showed that sequences of the two contigs present low similarity with proteins of sequenced genomes, showing that they derived from microorganisms whose genome sequences were not available. MEGAN analysis revealed that 16 and 17 ORFs respectively belonging to the clones 26H3 and 36A23 were assigned to the Bacteroidetes/Chlorobi group. None of the predicted genes in our contigs was assigned to a class, genus or species denoting that the two clones are originating from unidentified microorganisms.

**Fig 1:**
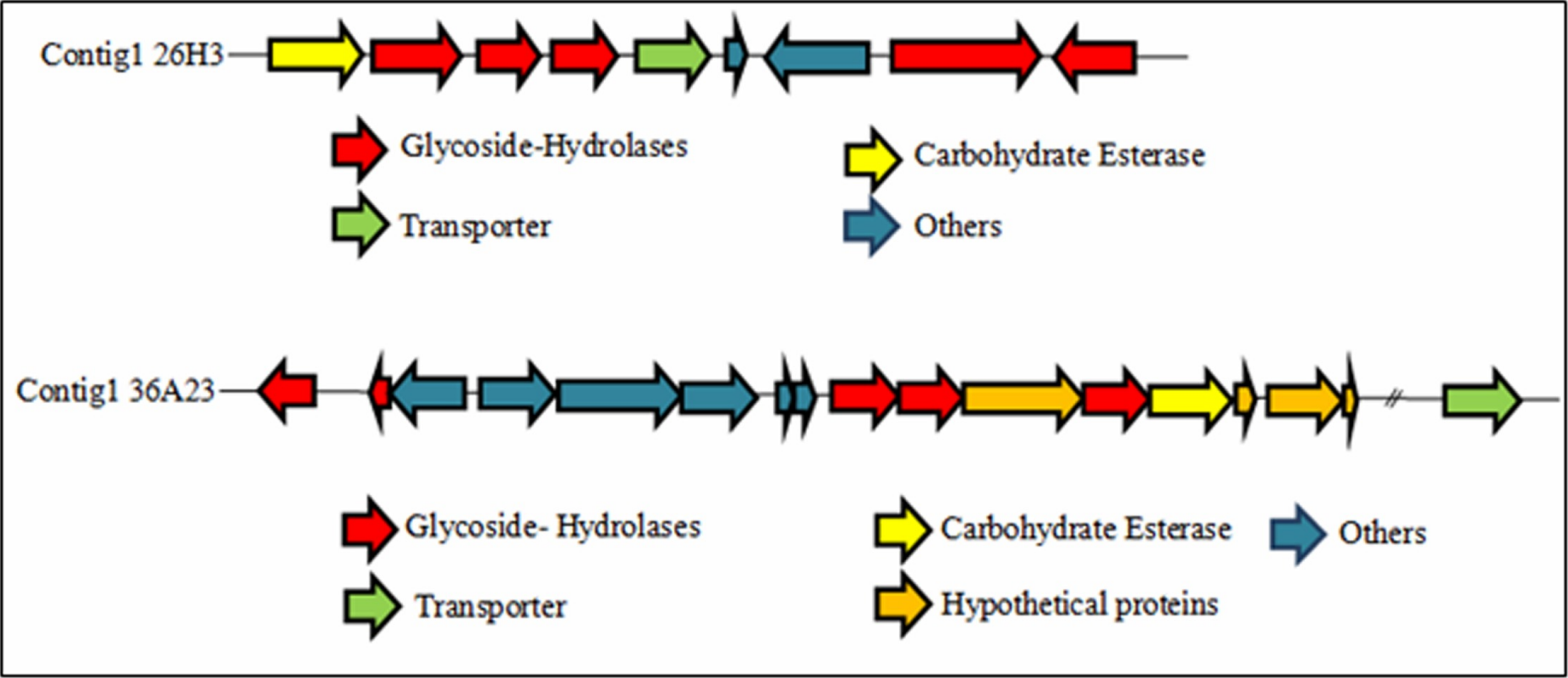
Metagenomic polysaccharide utilization loci showing organization of genes involved in the degradation and transport of plant cell wall polysaccharides in 26H3 and 36A23 clusters.

The functional annotation of 26H3 and 36A23 sequences highlighted gene clusters involved in the degradation and transport of plant cell wall polysaccharides (Tables 4 and 5, Fig 1).

**Table 4:** Predicted ORFs present on clone 26H3.

ORF N^o^	Contig position (nt)	RAST annotation of the non-CAZy putative proteins	Protein length (aa)	CAZy family of the best homologs
1	71–1657	-	528	CE7
2	1670–3214	-	514	GH5
3	3226–4320	-	364	GH26
4	4342–5499	-	385	GH130
5	5578–6921	Xyloside transporter XynT	447	-
6	7072–7254	Alpha galactosidase (EC 3.2.1.22)	60	-
7	9118–7244	FIG00402961: hypothetical protein	624	-
8	9333–11570	-	745	GH3
9	14130–11659	-	823	GH94
10	14671–14222	Transamidase GatB Domain protein	149	-
11	15742–14675	Mannose-1-phosphate Guanylyltransferase (GDP) (EC 2.7.7.22)	355	-
12	16025–15798	hypothetical protein	75	-
13	16603–16133	hypothetical protein	156	-
14	19214–16629	Recombination Inhibitory protein MutS2	861	-
15	20013–19216	Diadenylate cyclase spyDAC; Bacterial checkpoint controller DisA with nucleotide binding domain	265	-
16	20198–21370	putative purple acid phosphatase	390	-
17	21563–22186	LSU ribosomal protein L3p (L3e)	207	-
18	22190–22816	LSU ribosomal protein L4p (L1e)	208	-
19	22829–23119	LSU ribosomal protein L23p (L23Ae)	96	-
20	23132–23956	LSU ribosomal protein L2p (L8e)	274	-

**Table 5:** Predicted ORFs present on clone 36A23.

ORF N^o^	Contig position (nt)	RAST annotation of the non-CAZy putative proteins	Protein length (aa)	CAZy family of the best homologs
1	174–407	Hypothetical protein	77	-
2	461–946	Hypothetical protein	161	-
3	2649–1147	Cog1649 predicted glycoside hydrolase	500	-
4	3122–2667	probable integral membrane protein Cj0341c	151	-
5	3709–3125	Septum formation protein Maf	194	-
6	4896–3706		396	GH43 -
7	6099–4903	Aspartate aminotransferase (EC2.6.1.1)	398	-
8	6403–7761	Tryptophansynthase beta chain like (EC 4.2.1.20)	452	-
9	7773–9665	NAD synthetase (EC 6.3.1.5) / Glutamine amidotransferase chain of NAD synthetase	630	-
10	9686–11071	Glutamine synthetase type III, GlnN (EC 6.3.1.2)	461	-
11	11584–11751	Pyruvate flavodoxin oxidoreductase (EC 1.2.7.)	55	-
12	11760–12041	Pyruvate flavodoxin oxidoreductase (EC 1.2.7.)	93	-
13	12226–13284	-	352	GH26
14	13302–14270	-	322	GH5
15	14337–16172	FIG00402961: hypothetical Protein	611	-
16	16198–17172	-	324	GH16
17	17255–18616	-	453	CE1-CE6
18	18734–18961	Hypothetical protein	75	-
19	19264–20619	Hypothetical protein	451	-
20	20671–21309	Hypothetical protein	212	-
21	21436–21266	Hypothetical protein	56	-
22	21700–22377	Hypothetical protein	225	-
23	24992–22380	Excinuclease ABC subunit A paralog in greater Bacteroides group	870	-
24	25101–24973	Hypothetical protein	42	-
25	25222–28920	Phosphoribosyl formyl glycinamidine synthase, synthetase subunit (EC 6.3.5.3) / Phosphoribosyl formyl glycinamidine synthase, glutamine amidotransferase subunit (EC 6.3.5.3)	1232	-
26	28946–29968	putative sugar ABC transporter	340	-
27	30009–30182	Phosphoribosyl formyl glycinamidine synthase, synthetase subunit (EC 6.3.5.3) / Phosphoribosyl formyl glycinamidine synthase, glutamine amidotransferase subunit (EC 6.3.5.3)	57	-
28	30441–30659	Hypothetical protein	72	-
29	30656–31111	Hypothetical protein	51	-

The clone 26H3 was active on AZO-CM-Cellulose, AZCL-galactomannan and Tween 20. Its metagenomic insert was predicted to encode 7 genes for proteins being involved in polysaccharide degradation and transport. Based on their CAZy content (Table 4) and gene organization (Fig 1), we predict that this contig would consist of at least one locus involved in polysaccharide utilization, including ORF1 to ORF5 or ORF6. This locus would indeed contain one carbohydrate transporter (ORF5) and 4 CAZy encoding genes, ORF1, 2, 3, 4, of which the best blast hits belong to the CAZy families CE7, GH5, GH26 and GH130. All the characterized GH130 members are specific to mannosides. In addition, families GH5 and GH26 both include endo- and exo- acting enzymes specific to β-1-4-linked mannosides. Finally, family CE7 contains deacetylases acting on various substrates. Since mannans can be esterified in secondary cell walls [23], we predict that this locus would target acetylated plant mannans. Excluded from this locus, contig 26H3 also has two other genes (ORF8 and ORF9) with high homology with CAZy-encoding genes assigned to the families GH3 and GH94. These two families involve β-1,4-glucosidases and cellobiose-phsophorylases, respectively. ORFs 8 and 9 would thus explain the activity detected on modified celluloses.

The clone 36A23 was active on AZCL-Barley-β-Glucan and on AZCL-galactomannan. Its metagenomic insert was predicted to encode 7 genes coding for proteins involved in transport and degradation of the plant cell wall polysacharides and oligosaccharides. At the beginning of the contig, on the reverse DNA strand, there is a gene with no significant homology with genes encoding characterized CAZymes, but RAST- annotated as a glycoside hydrolase (ORF3), and a gene (ORF6) assigned to the GH43 family. Further on the locus, on the forward strand, we find CAZy-encoding genes assigned to families GH16, GH5, GH26 and CE6. These three GH families contain members acting on β-1,3–1,4-glucans and β-1,4 mannan, which explains the activities detected on these substrates. However, without characterizing the substrate specificity of each of these CAZymes, it is at this stage difficult to assign a specific hemicellulosic target substrate to this locus.

## Discussion

The dromedary is able to digest woody plant cell wall more efficiently than most other ruminants [2, 3]. Hence, we postulated that its digestive microbiota is very rich in a wide variety of enzymes that degrade lignocellulosic material. In the present work, we gained insight into the profile of the plant cell wall-degrading genes and hemicellulosic and cellulosic gene clusters in dromedary fecal microbiome which has never been achieved previously. Whence, we applied the metagenomic functional exploration to the dromedary digestive microbial population to discover new efficient enzymes applicable for animal feed and other biotechnological applications. High-throughput functional screening resulted in 47 hit clones. Two of them, harboring multiple activities, were retained for further analysis.

The taxonomic assignment revealed that most of predicted genes were assigned to the Bacteroidetes/Chlorobi group. This corroborates results of the bacterial diversity analysis of the dromedary fecal microbiota, based on analyzing the 16s rRNA gene previously reported [4, 6, 24] demonstrating that camel rumen metagenome is dominated by Bacteroidetes and Firmicutes. This denote that, as is other herbivores, bacteria related to those phyla are the predominant fiber degraders in rumen microbiota, producing a large number of plant cell wall degrading enzymes [25, 26]. The diversity of these enzymes in fibrolytic bacteria was confirmed in many studies by genome sequencing [27, 28]. In addition, gene profiles in dromedary fecal microbiota seems to be highly influenced by their diet including a broad spectrum of plants avoided by domestic ruminants like halophytes [5] and plants of poor digestibility such as shrubs and trees [29]. Ghanem and coworkers [30] revealed that the main residues in crude extract mucilage of halophytes were rhamnose, galactose, and glucose. In addition, the main monosaccharide detected in seeds of *Alhagi* species (camel thorn) is glucose, while mannose is dominated the epigeal part [31]. This would explain the high hit rate obtained on galactomannan and β-glucans, while no hit was obtained on xylan.

Functional annotation of 26H3 contig revealed predicted genes coding for proteins being involved in plant cell wall hydrolysis. We predict that 6 ORFs are included in a polysaccharide utilization locus to target acetylated mannans. Nevertheless, since ORF5 product is predicted to be a carbohydrate transporter from the MFS family, and thus does not present any significant similarity with SusC carbohydrate transporters that are the ‘labels’ of the bacteroidete Polysaccharide Utilization Loci (PULs) described in the PUL database (http://www.cazy.org/PULDB, [32]), we cannot name the present locus as a formal PUL. However, this locus presents a significant homology, in terms of CAZy content, with the predicted PUL 13 identified in *Dysgonomonas mossii* DSM 22836 (member of the Bacteroidetes/Chlorobi group) genome. They, nevertheless, differ in the order of genes in the locus. We thus suspect that SusC/D encoding genes could be present on the metagenomic locus discovered here, which would be truncated at the 5’ extremity in the 26H3 fosmid. Similar to 26H3 contig, the predicted PUL13 from *Dysgonomonas mossii* DSM 22836 also contains GH3 and GH94 encoding genes. Nevertheless, gene orientation in the 26H3 sequence indicates that the GH3 and GH94 encoding genes would not belong to the CE7-GH5-GH26-GH130 containing locus.

Functional annotation of the 36A23 contig revealed predicted genes coding for proteins being implicated in fiber degradation. Interestingly, the comparison of the 36A23CAZy gene cluster with Polysaccharide Utilization Loci (PULs) described in the PUL database revealed that it has no similarity with archived PULs.

In order to assess the originality of these metagenomics CAZymes, their sequences were compared to the amino acid sequences of characterized CAZymes derived from the CAZy database (Tables 6 and 7).

**Table 6:** Comparison of the sequences of 26H3 ORFS with uncharacterized and characterized proteins available in the CAZy and NCBI databases.

ORF	Most homologous uncharacterized protein (Blastp)				Most homologous characterized CAZyme), or protein encoded by a characterized locus			
	Protein	Cover (%)	Identity (%)	E-value	Protein	Cover (%)	Identity (%)	E-value
1	Hypothetical protein [*Bacteroides luti*] GenBank: WP_073399469.1	73	45	9e-109	cephalosporin C deacetylase [*Bacillus subtilis*] GenBank: AFP23353.1 (CE7) [33]	55	36	2e-44
2	uncharacterized protein BN702_00385 [*Bacteroides* sp. CAG:545] GenBank: CCZ44289.1	98	48	2e-165	β-glucanase [uncultured organism] GenBank: ADD61911.1 (GH5) [18]	93	45	8e-149
3	glycosyl hydrolase [unidentified microorganism] GenBank: CAJ19136.1	98	51	9e-134	SARM_0007 / 3811766_160700 / TW-10 [uncultured organism] GenBank: ADX05678.1 [34]	98	52	4e-139
4	conserved hypothetical protein [unidentified microorganism] GenBank: CAJ19137.1	99	82	0.0	β-1,4-mannosylglucose phosphorylase (MGP;BF0772) *Bacteroides fragilis* NCTC 9343 GenBank: CAH06518.1 (GH130) [35]	99	73	0.0
5	cation transporter [*Bacteroides intestinalis*] GenBank: WP_044533049	99	68	0.0	hypothetical protein [uncultured bacterium contig00010(2014)] GenBank: AIA99573.1 [36]	99	56	3e-173
8	beta-glucosidase [uncultured microorganism] GenBank: ADB80109.1	99	69	0.0	β-glucosidase (BglF3;Fjoh_0775) [*Flavobacterium johnsoniae* UW101] GenBank: ABQ03809.1 (GH3) [37]	96	43	0.0
9	Hypothetical protein [*Bacteroides uniformis*] GenBank: WP_016273975.1	100	83	0.0	Cellobionicacid phosphorylase 94B (Cep94B;CBAP;SdCBAP;Sde_0906) (Cep94B) (GH94) [*Saccharophagus degradans* 2-40] GenBank: ABD80580.1 [38]	97	70	0.0

**Table 7:** Comparison of the sequences of 36A23 ORFs with uncharacterized and characterized proteins available in the CAZy and NCBI databases.

ORF	Most homologous uncharacterized protein (Blastp)				Most homologous characterized protein (CAZy/Blastp), or protein encoded by a characterized locus			
	Protein	Cover (%)	Identity (%)	E-value	Protein	Cover (%)	Identity (%)	E-value
3	Hypothetical protein [*Bacteroides coprocola*] GenBank: WP_007571448.1	99	55	0.0	-	-	-	-
6	glycoside hydrolase [*Paludibacter propionicigenes*] GenBank: WP_013444857.1	99	67	0.0	endo-α-1,5-L-arabinanase (AbnZ2;Yxia1) [*Paenibacillus polymyxa*] GenBank: AHA50063.1 (GH43) [39]	40	25	5e-05
13	Hypothetical protein A2V46_15635 [Bacteroidetes bacterium RBG_19FT_COMBO_42_7] GenBank:OFY73025.1	98	57	3e-132	exo-β-1,4-mannanase (mannobiose-producing) (ManA;BfMan26;BF0771 [*Bacteroides fragilis* NCTC 9343] GenBank: CAH06517.1(GH26) [35]	98	42	5e-94
14	hypothetical protein [*Labilibacter aurantiacus*] GenBank: WP_068471806.1	98	53	3e-127	Fisuc_2933 / FSU_0196 [*Fibrobacter succinogenessub* sp. succinogenes S85] GenBank: ACX76513.1 (GH5-CBM4)	91	38	8e-61
16	glycoside hydrolase [marine bacterium AO1-C] GenBank: OJJ21706.1	83	53	4e-93	laminarinase (LamR) [*Rhodothermus marinus*] GenBank: AAC69707.1(GH16) [40]	77	49	2e-82
17	9-O-acetylesterase [Parabacteroides] GenBank: WP_005865579.1	99	48	3e-148	Acetylxylan esterase (R.17;R.17-1) [unidentified microorganism] GenBank: CAJ19109.1 (CE1-CE6) [41]	37	29	7e-08
26	putative multiple sugar transport system substrate-binding protein [*Prevotella ceaebacterium* HUN156] GenBank:SFW18185.1	99	66	1e-166	-	-	-	-

Genes assigned to the GH5, GH16 and GH26 families share less than 55% identity with their nearest characterized neighbors. Those assigned to the GH3, GH130 and GH94 families showed less than 75% identity with their nearest characterized exo-acting homologs. Finally, the CAZymes assigned to the CE7 and CE6 families displayed only 36% and 29% identity with characterized esterases. The most original putative CAZyme is that encoded by ORF3 of the clone 36A23, function. It has no significant similarity with members of known CAZy families. It could thus represent a member of a new CAZy family, but has low identity (55%) with its most homologous uncharacterized proteins in the NCBI NR database. Overall, the originality of the gene discovered in the present study could lead to the description of new structural features of prime interest for industrial biotechnologies.

## Conclusions

The present work constitutes the first activity-based metagenomic study targeting the dromedary gut microbiome. High-throughput screening results highlighted the high potential of dromedary fecal bacteria for the degradation of mannans and β-glucans. The detailed analysis of two original metagenomic loci belonging to bacteria from the Bacteroidetes/Chlorobi group revealed complex machineries involved in the metabolization of these major components of the wild dromedary diet. The in depth characterization of the specificity of each of these enzymes and transporters is the next challenge that will allow the understanding at the molecular level of the involvement of these loci in fiber degradation.
